# Design, Implementation, and Evaluation of an ADDIE Model‐Based Structured Educational Intervention to Improve Anesthesia Students' Patient Handover Skills: A Sequential Explanatory Mixed‐Methods Study

**DOI:** 10.1002/hsr2.72528

**Published:** 2026-05-13

**Authors:** Ali Khalafi, Golnoosh Rahimi‐Shandi, Nooshin Sarvi‐Sarmeydani

**Affiliations:** ^1^ Department of Anesthesiology, School of Allied Medical Sciences Ahvaz Jundishapur University of Medical Sciences Ahvaz Iran

**Keywords:** anesthesia, models, educational, patient handoff, students, nursing

## Abstract

**Background and Aims:**

Patient handover from the operating room to the recovery unit is critical but error‑prone in perioperative care. Despite its importance, structured training in handoff communication is rarely included in anesthesia curricula. This study aimed to design and evaluate an ADDIE‑based educational intervention to improve anesthesia students' handover competencies.

**Methods:**

A sequential explanatory mixed‐methods design was employed, with a qualitative phase followed by a quantitative RCT (pre‐test/post‐test control group). Educational needs were explored via semi‐structured interviews with anesthesia students, instructors, and recovery staff, plus clinical observation. Interview data underwent conventional content analysis based on the approach described by Hsieh and Shannon (2005). Based on these findings, a two‐session intervention was developed, incorporating simulation‐based role‐playing, video analysis, and multi‐source feedback. Sixty‐three undergraduate anesthesia students were individually randomized to intervention (*n* = 32) or control (*n* = 31) using a random numbers table, with semester as stratification variable. Handover skills were assessed pre‐ and post‐training using a validated 13‐item checklist; data were analyzed with paired/independent *t*‐tests.

**Results:**

The intervention and control groups were comparable at baseline (3.34 vs. 3.03, *p* = 0.343). Both groups improved, but the intervention showed a significantly greater increase in handoff skills, with mean scores rising from 3.34 ( ± 1.47) to 8.25 ( ± 2.24) (*p* < 0.001, within‐group Cohen's *d* = 1.692). The control group's improvement was modest (from 3.03 ± 1.08 to 3.61 ± 0.91). The between‐group comparison confirmed the superiority of the ADDIE‐based intervention (mean difference: 4.623, 95% CI: 3.741–5.505, *p* < 0.001). Structured theory with active learning effectively translated knowledge into practical competency.

**Conclusion:**

The ADDIE‑based intervention significantly enhanced handoff skills, highlighting the value of systematic instructional design in clinical education. These findings support integrating standardized communication training into curricula and warrant future multi‑center studies with long‑term follow‑up.

## 1 Introduction

Patient handover from the operating room to the recovery unit is a critical point in perioperative care, where accurate transfer of information directly influences patient safety and outcomes [1, 2, 3]. This involves communication of key patient information, such as intraoperative events, anesthetic management, and hemodynamic stability, which must be transferred to recovery room staff [2, 3]. The handover forms a transition between two very different care settings and necessitates attention to details and protocols of structured communications to ensure continuity of care. However, this most critical transitional period is identified to represent a weak point in the delivery of care to patients. Communication breakdowns and lapses in information can result in adverse events, delayed complication recognition, and compromised safety for the patient [4].

Adequate patient handover is of utmost importance, as poor communication during this sensitive transfer has been related to medication errors, missed clinical deterioration, delayed interventions, and increased morbidity and mortality rates. The Joint Commission has reported that two‐thirds of adverse events result from communication errors and more than 50% of these occur during handover by healthcare providers [5, 6]. The difficulty in the management of anesthesia, with patients in an acutely changing physiological state during emergence from anesthesia, demands an appropriate and comprehensive flow of information. Recovery also requires close monitoring for potential complications like respiratory depression, hemodynamic instability, postoperative nausea and vomiting, pain management problems, and residual neuromuscular blockade, which all require full knowledge of intraoperative events and patient‐specific risk factors [7, 8]. Under these high‐stakes circumstances, standardized, evidence‐based practices for handover are not a matter of professional efficiency but a basic imperative in patient safety, requiring immediate attention and systematic intervention.

Despite the well‐documented importance of structured handover processes in optimizing patient outcomes, there is a significant gap in the formal education and training of healthcare professionals, particularly anesthesia students and residents, with respect to standardized patient handover protocols [9]. Current medical and nursing curricula seldom provide specific, comprehensive modules on the acquisition of handover communication skills, forcing practitioners to learn these critical competencies through inconsistent experience‐based approaches. This educational deficiency is particularly pronounced in anesthesia training programs, where technical and pharmacological aspects of care have received greater emphasis than communication competencies and handover procedures [10]. The absence of structured educational interventions has led to considerable variability in handover quality, with practitioners often developing idiosyncratic approaches that may omit critical information or fail to follow best‐practice guidelines [9].

The present study utilized the ADDIE (Analysis, Design, Development, Implementation, and Evaluation) instructional design model to systematically develop, implement, and evaluate an educational intervention. The ADDIE model is a systematic instructional design framework that provides a structured approach to curriculum development through five iterative phases: Analysis (identifying learning needs), Design (defining objectives and planning), Development (creating educational materials), Implementation (delivering the intervention), and Evaluation (assessing outcomes). This model was chosen because it ensures that the intervention is grounded in a thorough needs analysis, aligns instructional strategies with identified gaps, and includes built‐in evaluation to measure effectiveness—features often lacking in traditional curriculum development approaches in medical education [11, 12]. To our knowledge, no previous study has applied the ADDIE model to develop a handoff training program specifically for anesthesia students within a randomized controlled trial. The novelty of this study is the systematic use of this established education framework to create a structured, theoretically‐informed curriculum specifically tailored to the unique needs of operating room to recovery handover. The aim of this study was to design, implement, and evaluate an ADDIE‐based structured educational intervention to improve patient handover skills from the operating room to the recovery unit among anesthesia students. We hypothesized that the intervention group would demonstrate significantly higher handover skills compared to the control group after training.

## 2 Materials and Methods

### 2.1 Study Design and Setting

This study employed a sequential explanatory design consisting of a qualitative phase to inform the development of the intervention, followed by a quantitative randomized controlled trial with a pre‐test/post‐test control group. It was conducted at Ahvaz Jundishapur University of Medical Sciences (AJUMS), Iran, during the academic period from November to December 2025. Data collection and interventions took place in the meeting room and practical skills laboratory of the School of Allied Medical Sciences and the operating rooms of affiliated teaching hospitals in Ahvaz.

### 2.2 Study Participants and Sampling

Sampling method: A multistage convenience sampling method was used to recruit participants. Sixty‐three undergraduate anesthesia students in their fifth and seventh semesters who met the inclusion criteria were enrolled.

**FIGURE 1:**
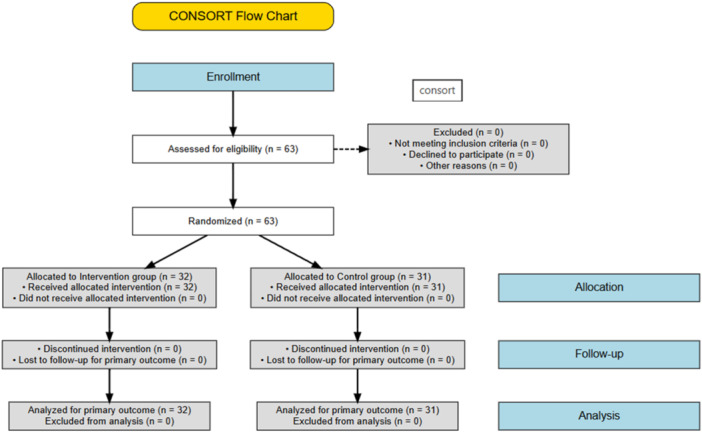
CONSORT flow diagram of participant recruitment, allocation, and analysis.

Random assignment: Participants were then randomly assigned to the intervention (*n* = 32) or control (*n* = 31) group using a table of random numbers, with academic semester as a stratification variable to ensure balanced allocation. Allocation concealment was ensured by using sequentially numbered, opaque, sealed envelopes, which were opened only after participant enrollment (Figure 1).

Inclusion criteria were: being a fifth or seventh‐semester undergraduate anesthesia student, willingness to participate in the research, and full cooperation in the intervention.

Exclusion criteria applied before enrollment were: concurrent participation in another study or training session focused on pre‐anesthesia care.

Withdrawal criteria applied after randomization included: absence from more than one intervention session, non‐participation in the pre‐test or post‐test, withdrawal from the study at any time for any reason, or incomplete completion of study questionnaires.

An a priori power analysis was conducted using G*Power software based on parameters from prior similar research [13]. Assuming a large effect size (Cohen's *d* > 0.8) as reported in previous handoff training studies, with *α* = 0.05 and power = 0.80, the required sample size was estimated to be 26 participants per group (total 52). To account for potential attrition, we recruited 63 students, which exceeded the minimum required sample size.

To minimize the risk of contamination between groups, all participants were explicitly instructed not to discuss the training content or share any materials with others until the study was completed.

### 2.3 Data Collection Tools and Techniques

The data collection instruments in this study consisted of two researcher‐developed components designed based on a review of the relevant literature and expert interviews. The first component was a demographic questionnaire that collected basic student information, including sex, age, cumulative GPA, and academic semester. These data were used to describe the characteristics of the sample and to control for background variables in the analyses.

While standardized handover tools such as SBAR, I‐PASS, and PHAT are well‐established for general clinical communication, they are not specifically designed for the operating room to recovery unit handover in anesthesia education. This context requires additional information not typically captured by these tools, including type of anesthesia, intraoperative fluids, blood products administered, and specific recovery concerns. Therefore, we developed a context‐specific 13‐item checklist tailored to the unique needs of anesthesia handover. This checklist was conceptually informed by the ISBAR (Identification, Situation, Background, Assessment, and Recommendation) framework, an internationally recognized handover communication tool, while being adapted and expanded to include anesthesia‐specific items relevant to the operating room to recovery context. Each item was scored dichotomously (0 = not performed, 1 = performed), with a maximum possible score of 13. The checklist items were organized into four domains: patient demographic information, surgical and anesthesia details, clinical status and patient monitoring, and medication management with postoperative care. Content validity of the instrument was assessed by a panel of 10 experts, including six anesthesia specialists and four faculty members from the Department of Anesthesia. To ensure practical relevance, the checklist was also reviewed by two experienced PACU nurses for feedback on clinical applicability. Each item was rated by all experts for relevance and clarity using a 4‐point scale. Based on their feedback, three items were reworded for clarity, and one item was removed due to redundancy. The content validity index (CVI) was 0.83, and the content validity ratio (CVR) was 0.86. Face validity was assessed by 15 anesthesia students from the target population (outside the main study sample), who confirmed that the items were clear, relevant, and understandable. Necessary revisions were applied. Reliability of the checklist was examined by administering it to 10 students outside the intervention group. Internal consistency (KR‐20) was 0.78. Inter‐rater reliability, assessed using Cohen's kappa, was 0.72, and test–retest reliability over a 10‐day interval was 0.83. Outcome assessors were blinded to group allocation throughout the study. All items were equally weighted, with a total possible score of 13. No cut‐off score was predefined, as the checklist was used to compare group means rather than to classify individual competence.

#### 2.3.1 Control Group

Students in the control group received standard clinical internship training, which followed a traditional apprenticeship model. Learning occurred opportunistically based on daily surgical cases, and informal instruction on patient handover was provided by supervising instructors during routine activities. The training was unstructured and varied considerably depending on the clinical supervisor and surgical schedule, with no dedicated sessions or standardized content on patient handover.

### 2.4 Research Stages

#### 2.4.1 Stage 1: Analysis

During the analysis stage, a multi‐source assessment was conducted to identify the educational gaps in anesthesia students' handoff procedures from the operating room to the recovery unit. Data collection was accomplished through three main methods:

- Direct observation of students' performance in the clinical setting.
- Review of common errors observed during the process of patient handoff.

Direct observations were conducted for 10 clinical handovers over a 2‐week period, each lasting approximately 5 min. Observations were recorded using a field note protocol, and common errors were documented systematically based on a predefined checklist of handover steps.

Semi‐structured interviews were conducted with three key stakeholder groups: anesthesia students, clinical instructors, and recovery room staff.

Inclusion criteria for interviews were:

a. for students: being in the fifth or seventh semester and having completed at least one clinical rotation in the operating room;
b. for instructors: having at least 4 years of clinical teaching experience in anesthesia;
c. for recovery staff: having at least 5 years of experience in the PACU.

Exclusion criteria were unwillingness to participate or inability to attend the interview session.

Interviews were conducted with 12 participants (four anesthesia students, four clinical instructors, and four recovery room staff), each lasting approximately 15 min. Participants were selected using purposive sampling to ensure diverse perspectives. Interviews were conducted in a private room at the clinical setting, audio‐recorded, and transcribed verbatim. All interviews were conducted by the first author. Data collection continued until thematic saturation was reached, meaning no new themes emerged from subsequent interviews. Data were analyzed using conventional content analysis based on the approach described by Hsieh and Shannon (2005), with two researchers independently coding the data. Disagreements were resolved through discussion to achieve consensus, and inter‐coder reliability was assessed. To enhance the trustworthiness of the qualitative findings, several strategies were employed. Member checking was conducted by sharing the summarized findings with three participants (one from each stakeholder group) to confirm the accuracy of interpretations. Peer debriefing was performed with an experienced qualitative researcher who reviewed the coding process and themes. Additionally, independent coding by two researchers and data saturation further strengthened the credibility and dependability of the analysis.

The five key educational gaps identified through the thematic analysis—ambiguity in the handoff process, incomplete information, deficiencies in clinical skills, psychological and educational challenges, and the need for structured training and simulation—directly informed the design of the educational intervention described in the following stages (Table 1).

**TABLE 1:** Qualitative findings from the educational needs assessment.

Main theme	Subthemes	Sample quotes
Ambiguity in the patient handoff process	Lack of knowledge about essential information, confusion in prioritization, and difficulty in documentation or verbal delivery	*“I never know exactly what information is essential for patient handoff.” (Student 1)*
		*“Sometimes I don't know what information should be transferred during handoff.” (Student 10)*
Incomplete information and threats to patient safety	Providing incomplete information, forgetting critical details, and potential risk to patient safety	*“The information I receive is not complete, and I cannot make the right decision.” (Student 4)*
		*“Students often provide incomplete information, which can lead to clinical errors.” (Instructor 1)*
Deficiencies in clinical skills	Difficulty in rapid assessment of vital signs, inability to describe patient status, and need for more practice	*“I don't have the skill to quickly assess vital signs, and it is stressful.” (Student 6)*
		*“Students lack sufficient skills in assessing vital signs and need more practice.” (Instructor 3)*
Psychological and educational challenges	Fear of mistakes, high stress, lack of self‐confidence, and limited opportunities for guidance	*“I don't have enough self‐confidence to manage the handoff.” (Student 16)*
		*“I feel a lot of stress during patient handoff and forget some important points.” (Student 7)*
Need for structured training and simulation	Request for real scenarios, need for standardized training, and importance of organized educational programs	*“If we practice with a real scenario, I can transfer information more accurately.” (Student 12)*
		*“A structured educational program based on a scientific model can improve students' skills and confidence.” (Instructor 7)*

For example, the theme “ambiguity in the handoff process” highlighted the need for clear, structured guidelines, which were incorporated into the video analysis session. “Deficiencies in clinical skills” and “psychological challenges” underscored the importance of hands‐on practice and peer support, leading to the inclusion of role‐playing and structured feedback in the second session. Thus, the qualitative findings ensured that the intervention was tailored to the specific needs identified by stakeholders.

This triangulation approach provided a comprehensive view of the learning gaps among the students and formed the basis for subsequent design in the educational intervention.

#### 2.4.2 Stage 2: Design

Based on the findings from the analysis stage, the structure of the educational intervention was designed to address two key objectives: enhancing verbal handoff reporting skills and improving professional communication. The intervention was structured as two consecutive training sessions, incorporating a combination of theoretical instruction, group‐based analysis, role‐playing in a simulated environment, and multi‐layered feedback.

The intervention was designed based on Kolb's experiential learning theory, which emphasizes a cycle of concrete experience, reflective observation, abstract conceptualization, and active experimentation. To maintain theoretical fidelity, the concrete experience (CE) phase occurred at the beginning of the first session, where each student performed an initial simulated handover without prior instruction. This was followed by video analysis and group discussion, which served as the reflective observation (RO) phase. Accordingly, the first session lasted 90 min and focused on theoretical instruction and reflective observation through video analysis of handover scenarios. The second session lasted 120 min to allow sufficient time for active experimentation through role‐playing, simulation, and structured feedback from instructors and peers. This structure enabled learners to progress through the experiential learning cycle while accommodating the practical demands of each session.

#### 2.4.3 Stage 3: Development

The educational resources were designed and compiled in collaboration with, and following approval by, faculty members in the Department of Anesthesia and board‐certified anesthesiologists during the development phase. Resources included:

- Instructional slides: Well‐organized per key and challenging concepts with visual aids to enhance understanding
- Educational video clips: These are less than 10‐min‐long video clips with samples of good and bad patient handoff practices that can be accessed through mobile phones anywhere and anytime. For procedural steps, however, instructors need to demonstrate them in person.

The educational videos were developed using simulated handoffs performed by master's anesthesia students who had at least 1 year of teaching experience and 2 years of clinical experience. A total of 10 videos were created: five demonstrating effective handoffs and five illustrating common errors. The complexity of the scenarios was standardized by expert review. Although the videos were designed to be accessible for self‐directed learning, in this study they were both shown in class for structured group discussion and made available to students for individual review.

- Simulated scenarios: Based on common clinical scenarios, including information about the patient, type of surgery, current status, and potential challenges during handoff.
- Feedback framework: Structured to provide verbal and systematic feedback by instructors and peers. After each simulation, structured feedback was provided by the instructor and peer students based on key criteria, including accuracy, sequencing, safety, and communication.

#### 2.4.4 Stage 4: Implementation

The educational intervention was conducted over two consecutive sessions.

##### 2.4.4.1 Session 1: Theoretical Instruction and Group Analysis

At the beginning of the session, each student performed an initial simulated handover without prior instruction. This served as the concrete experience (CE) phase of Kolb's experiential learning cycle. Core concepts on safe and effective patient handoff were taught by the instructor in the first session. Students then viewed selected educational video clips illustrating both successful and deficient handoff practices. A total of 10 video vignettes (five effective and five with common errors) were shown and analyzed. To guide the analysis, students were provided with a structured checklist that included key criteria such as accuracy of information, logical sequencing, attention to patient safety, and quality of professional communication. Students discussed their observations in small groups before sharing with the whole class. After viewing each clip, the students analyzed the strengths and common errors of the handoff, along with the clinical implications in groups. This stage was intended to trigger prior knowledge, enhance critical thinking, and prepare the students mentally for the practical aspect.

##### 2.4.4.2 Session 2: Simulation Environment Role‐Playing

In this session, each student received a randomly selected clinical scenario card and engaged in simulated handoffs as members of the anesthesia team. Each team presented a verbal handover within the given time. The instructor played the role of recovery room staff and added dynamic elements such as clinical questions or altered patient status to enhance students' decision‐making, prioritization, and communication.

The first session lasted 90 min and the second session lasted 120 min, with a 1‐week interval between sessions to allow for reflection.

During the session, students worked in rotating roles. In each round, one student performed the handoff as the anesthesia provider, while another student was assigned to provide structured feedback immediately after the simulation. Following the performance, all remaining students and the instructor also provided feedback based on the predefined checklist criteria (accuracy, sequencing, safety, and communication). Multiple rounds were conducted, with teams reorganized and roles rotated in each round, so that every student had the opportunity to perform the handoff at least once, serve as the designated peer feedback provider, and observe others. This structure ensured active participation, multiple perspectives, and reinforced learning through peer assessment and reflection.

###### 2.4.4.2.1 Multilevel Feedback: Instructor and Peer Review

Feedback was provided immediately after each simulation, with approximately 5–7 min per performance, following a structured approach based on the checklist criteria. The instructor facilitated the process, inviting peer feedback first, then other students, and concluding with their own summary, while encouraging the performer to reflect.

Moreover, observer students engaged in peer feedback, which included reviewing their classmates' performances. They identified strengths, missing or unclear elements, and provided constructive suggestions for improvement. Such collaborative engagement encouraged reflective learning among peers along with deeper understanding of the standards for safe handoffs.

#### 2.4.5 Stage 5: Evaluation

The post‐test was conducted 1 week after the intervention during the students' routine clinical clerkships and internships in the affiliated teaching hospitals. Patient handover is part of the students' standard clinical responsibilities; therefore, assessments were integrated into their regular duties under the continuous and direct supervision of both clinical instructors and the attending anesthesiologist accompanying the patient. Supervisors were present at all times and authorized to intervene immediately if any aspect of patient safety was compromised. To ensure standardization despite varying case complexity, assessments were based on the same 13‐item checklist focusing on the handover process rather than case‐specific details.

All assessments were conducted by two trained independent evaluators who were blinded to group allocation, using the same checklist. The pre‐test was conducted 1 week before the intervention under identical conditions.

### 2.5 Ethical Considerations

This study was approved by the Ethics Committee of AJUMS (IR.AJUMS.REC.1404.185) and performed with adherence to the principles of the Declaration of Helsinki (2013). The students were thoroughly informed about the study goals, procedures, conditions concerning participation, the right to withdraw at any stage without any negative consequences, and the strict confidentiality of their data. Afterward, written informed consent was obtained from the students who agreed to participate in the study. In addition to consent for the trial, informed consent was obtained from all participants for the qualitative components, including interviews and direct observations. Participants were assured that their data would remain confidential and that they could withdraw at any time without consequences.

### 2.6 Statistical Analysis

Qualitative analysis: Qualitative data were analyzed using conventional content analysis (Hsieh & Shannon, 2005) with the assistance of NVivo software, version 14. The analysis process was conducted independently by two researchers. Disagreements were resolved through discussion to achieve consensus, and inter‐coder reliability was assessed to enhance the rigor of the findings.

Quantitative analysis: Quantitative data were analyzed using SPSS software, version 26. The normality of quantitative variables was assessed using the Shapiro–Wilk test. Standard statistical methods were used as described in Pagano and Gauvreau [14]. Comparisons of pre‐test and post‐test scores within groups were performed using the paired‐samples *t*‐test, and comparisons between groups were made using the independent‐samples *t*‐test. Fisher's exact test was used to determine any significant differences between the two groups for categorical variables, and McNemar's test was employed to assess changes between pre‐test and post‐test measurements for these variables. Partial eta squared (*η*²) was used as a measure of effect size for the ANCOVA, and Cohen's *d* was used to calculate effect sizes for within‐group paired comparisons. For all the tests, the level of significance was set at a *p*‑value < 0.05, and all tests were two‐tailed. An a priori power analysis was conducted using G*Power software, confirming that the sample size (*n* = 63) was adequate to detect large effects with 80% power at *α* = 0.05.

## 3 Results

### 3.1 Comparison of Demographic and Baseline Variables

The demographic and academic baseline characteristics were compared between the intervention and control groups.

The total sample consisted of 63 undergraduate anesthesiology students, divided into an intervention group (32 students) and a control group (31 students). In the intervention group, there were 17 (53.1%) females, and 15 (46.9%) males, while in the control group, there were 15 (46.9%) females and 16 (51.6%) males. The Chi‐square test showed no significant difference in the sex distribution of both groups (*p* = 0.803).

Distribution across academic semesters was also equal, with 15 (46.9%) students in the fifth semester and 17 (53.1%) in the seventh semester in the intervention group. Similarly, there were 15 (48.4%) students in the fifth semester and 16 (51.6%) in the seventh semester in the control group. Based on the Chi‐square test, there was no statistical difference in the semester distribution between the groups (*p* = 0.892) (Table 2).

**TABLE 2:** Baseline characteristics of participants by study group.

Variable	Category	Control (*n* = 31)	Intervention (*n* = 32)	*p* value
Age (mean ± SD)		22.42 ± 1.76	22.19 ± 1.53	0.580
GPA (mean ± SD)		18.35 ± 0.95	18.34 ± 0.98	0.950
Sex, *n* (%)	Female	15 (48.4)	17 (53.1)	0.803
	Male	16 (51.6)	15 (46.9)	
Academic semester, *n* (%)	5	15 (48.4)	15 (46.9)	0.892
	7	16 (51.6)	17 (53.1)	

*Note: p*‐values for age and GPA were calculated using the independent samples *t*‐test; *p*‐values for sex and academic semester were calculated using the Chi‐square test. All tests were two‐tailed.

The mean age was comparable between the intervention (22.19 ± 1.53 years) and control groups (22.42 ± 1.77 years). An independent samples *t*‐test confirmed that this difference was statistically non‐significant (*p* = 0.580).

The mean GPA in the intervention group was 18.34 ± 0.984, and in the control group it was 18.35 ± 0.955. Also, no significant difference was observed between the two groups (*p* = 0.950) (Table 2).

In all, statistical analyses confirmed that the intervention and control groups were equal with regard to all demographic and academic baseline variables.

### 3.2 Comparison of Pre‐Test Results Between Intervention and Control Groups

The results of the pre‐tests of both groups showed about the same levels. In the intervention group, the mean overall pre‐test score was 3.34, with that of the control group being 3.03. This difference is not significant (*p* = 0.343). These results show that the participants of both groups arrived at the beginning of the study with approximately equal basic skills.

### 3.3 Comparison of Intervention and Control Group Outcomes

As shown in Table 3, both groups demonstrated significant improvements after training (*p* < 0.001).

**TABLE 3:** Mean scores of skill level regarding patient handover from operating room to recovery in the control and intervention groups.

Group	Pre‐test (mean ± SD)	Post‐test (mean ± SD)	Mean difference (post–pre)	*p* value
Control	3.03 ± 1.08	3.61 ± 0.91	0.58	< 0.001
Intervention	3.34 ± 1.47	8.25 ± 2.24	4.91	< 0.001
Between‐group			4.623	< 0.001

*Note:* Within‐group comparisons were performed using the paired *t*‐test. The between‐group comparison was performed using ANCOVA with pre‐test scores as the covariate. Effect sizes are reported as Cohen's *d* with 95% confidence intervals.

#### 3.3.1 Control Group

The mean handoff skill score increased from 3.03 ± 1.08 at pre‐test to 3.61 ± 0.92 at post‐test. The paired *t*‐test showed a significant improvement (*t*(30) = −4.811, *p* < 0.001), with a moderate effect size (Cohen's *d* = 0.864, 95% CI: 0.445–1.273).

#### 3.3.2 Intervention Group

The mean handoff skill score increased significantly from 3.34 ± 1.47 at pre‐test to 8.25 ± 2.24 at post‐test (paired *t*‐test: *t*(31) = −9.570, *p* < 0.001), with a large effect size (Cohen's *d* = 1.692, 95% CI: 1.142–2.230).

To directly compare the post‐intervention handoff skills between the two groups while controlling for baseline differences, a one‐way analysis of covariance (ANCOVA) was conducted with pre‐test scores as the covariate. The results revealed a statistically significant difference between the intervention and control groups (F(1, 60) = 109.823, *p* < 0.001, Partial *η*² = 0.647), indicating a large effect size. After adjusting for pre‐test scores, the estimated marginal mean for the intervention group (8.243, SE = 0.308) was significantly higher than that of the control group (3.620, SE = 0.313). The mean difference between groups was 4.623 (95% CI: 3.741–5.505, *p* < 0.001). These findings confirm that the ADDIE‐based educational intervention was significantly more effective than traditional training in improving patient handover skills.

## 4 Discussion

The present study showed that an educational intervention, designed and evaluated using the ADDIE instructional design model and enriched with role‐playing and peer review, significantly improved anesthesia students' patient handoff skills from the operating room to the recovery unit. This finding confirms the increasing body of evidence suggesting that systematic and learner‐centered frameworks are an imperative for the efficient development of curricula in medical education. Specifically, the ADDIE model, with its emphasis on initial learner needs analysis and continuous evaluation, seems to be crucially important in achieving these educational outcomes. Thus, the model guaranteed that not only were the instructional strategies standardized but also precisely directed at the identified gaps in the competencies of patient handoffs. This finding supports Santos et al. (2019), who stated that nursing education based on theoretical and learner‐centered models promotes professional practice [15]. Our results expand this view by demonstrating that an ADDIE‐based intervention, in synergistic interaction with active learning strategies, can lead directly to improvements in clinical communication and patient safety.

However, it is important to interpret these findings within the context of the broader literature, which also identifies methodological heterogeneity and mixed outcomes in handover and communication training. A recent systematic review of surgical handover interventions, conducted by Ryan et al. (2024), reported that although many studies show improvements in process and staff outcomes, there is significant variability in methods, outcomes assessed, and overall evidence quality, limiting the ability to draw definitive conclusions about best practices [16]. Our study extends this evidence by demonstrating that a structured, theory‐driven ADDIE‐based intervention can enhance clarity and consistency in handover training.

Evidence from diverse educational contexts increasingly substantiates the ADDIE model as a robust instructional framework. For instance, Luo et al. (2024) showed that blended learning based on ADDIE improved nursing staff's theoretical knowledge and practical skills, self‐directed learning, and teaching satisfaction [11]. Similarly, Zhang et al. (2024) concluded that the ADDIE model, in combination with flipped classrooms, was an effective neurology education strategy that not only improved the theoretical and practical competencies of residents but also enhanced their independent learning and critical thinking abilities [17]. These findings align with our experience, whereby the structured design and evaluation phases of the ADDIE model enabled our intervention to be both context‐specific and outcome‐oriented. Nevertheless, not all educational interventions yield consistent benefits; for example, quasi‑experimental studies of communication training among health professions students show variable translation of improved communication knowledge into consistent error communication competence [18]. We argue that the initial analysis phase was particularly critical, allowing the precise identification of weaknesses in patient handoffs from the operating room to the recovery unit, therefore facilitating the customized design and development of role‐playing scenarios that directly addressed these deficiencies.

The active learning strategies inherent in our intervention, especially role‐play and peer review, acted as a linchpin in connecting the dots between theoretical knowledge and practical competency. The synergy between the ADDIE framework and interactive pedagogical methods appears to have been a primary driver of the study's positive outcomes. While the ADDIE model provided the systematic structure for curriculum design, the inclusion of role‐playing and peer review facilitated the transition from theory to mastery. These observations are consistent with research by Xu et al. (2023), who demonstrated that peer role‐playing significantly enhances clinical skills, communication, and professionalism compared to traditional bedside instruction [19]. Similarly, Larti et al. (2018) reported that role‐playing interventions enhanced empathy among nursing students in the operating room, bringing into light the enhancement of interpersonal competencies through experiential learning [20]. In our study, role‐playing allowed participants to practice and internalize the process of patient handoff safely, while peer review enhanced the critical observation skills of participants and provided constructive feedback immediately, thereby reinforcing self‐efficacy and collaborative learning. This result is in agreement with the report by Krishnan et al. (2020), where simulation‐based handoff training improved learners' understanding of perioperative communication and reduced knowledge gaps [21].

The improvements in patient handoff skills observed in the present study contribute to the greater goal of improving patient safety. Kaware et al. (2025) illustrated that education significantly enhanced nurses' perceptions of handoff processes and a culture of safety in hospitals in Nigeria [22]. Our results imply that the integration of ADDIE‐based instructional design, combined with role‐playing and peer review, serves to enhance not only the clarity and competence of communication among students but also, potentially, reduces information omission and technical errors associated with patient transitions.

It is also noteworthy that the control group showed a modest but statistically significant improvement (from 3.03 to 3.61, *p* < 0.001). This may be attributed to the effect of repeated exposure to clinical handovers during their routine internships, or to a possible Hawthorne effect. This conventional training itself may have contributed to their modest improvement, even without receiving the structured ADDIE‐based intervention. However, the magnitude of improvement in the intervention group was substantially larger, as confirmed by the ANCOVA results.

To ensure the rigor of the qualitative findings, Lincoln and Guba's framework was used to ensure the quality and robustness of the findings [23]. Several methods were used to enhance data credibility, including the accurate recording and full transcription of interviews, participant review and confirmation of the interpretations, as well as the evaluation of the initial categorizations by one anesthesia specialist together with two faculty members from the Department of Anesthesia. Dependability was enhanced by having the interview coding repeated by a co‐author who had substantial experience in qualitative data analysis. Further, all stages and details of the research were documented in full to enable external auditing and verification.

### 4.1 Limitations and Recommendations

Despite these encouraging findings, a number of limitations in this study need to be taken into account: this was a single‐university sample, and thus generalizability of findings may be limited. Due to the educational nature of the intervention, blinding of participants was not possible; however, the evaluators of student performance were blinded to group allocation. Skill assessments occurred immediately and 1 week post‐intervention without long‐term follow‐up to determine if improved handoff skills would be sustained over time. Additionally, although participants were instructed not to share information, the possibility of contamination between the intervention and control groups cannot be completely ruled out, as all students were from the same academic program. This may have slightly diluted the observed intervention effect. Furthermore, the use of a researcher‐developed checklist, although rigorously validated, limits the direct comparability of our findings with studies that employed internationally standardized tools such as PHAT or I‐PASS.

To strengthen external validity, future investigations should employ multicenter designs and larger sample sizes, ensuring the findings are generalizable across diverse clinical and educational settings. Long‐term follow‐up measurements are also needed to determine whether skill acquisition is durable over time. Further research should also examine whether improvements in handoff skills directly translate into better patient safety outcomes and how innovative technologies may enhance learning efficacy. Lastly, future research needs to examine whether this type of education actually improves patient outcomes, such as fewer errors and improved patient safety, in order to afford more robust evidence of clinical efficacy.

## 5 Conclusion

This study demonstrates that a structured educational intervention, systematically developed through the ADDIE instructional design model, led to significant improvements in the handoff proficiency of anesthesia students during the critical transition from the operating room to the recovery unit. This intervention effectively integrated active learning strategies like role‐playing, simulation, and peer feedback into the learning environment that bridges the gap between theoretical knowledge and clinical practice. These findings suggest that incorporating standardized communication training into anesthesia curricula may contribute to patient safety by improving handover competencies. Despite being limited to a single university and short‐term evaluation, the results strengthen the evidence that systematic application of learner‐centered and evidence‐based instructional frameworks can meaningfully impact improvement in clinical education. Such results may form a basis for future development of educational policies in health professions training. Overall, these findings highlight the pivotal role of structured instructional design models such as ADDIE in bridging the gap between theory and practice, ultimately contributing to the potential enhancement of patient safety and clinical education.

## Author Contributions

**Ali Khalafi:** conceptualization, supervision, writing – review and editing, project administration, validation. **Golnoosh Rahimi Shandi:** writing – review and editing, data curation, writing – original draft, investigation, resources. **Nooshin Sarvi Sarmeydani:** supervision, project administration, writing – review and editing, conceptualization.

## Disclosure

The lead author Golnoosh Rahimi Shandi affirms that this manuscript is an honest, accurate, and transparent account of the study being reported; that no important aspects of the study have been omitted; and that any discrepancies from the study as planned (and, if relevant, registered) have been explained.

## Conflicts of Interest

The authors declare no conflicts of interest.

## Policy on Using ChatGPT and Similar AI Tools

During the preparation of this work, the authors used ChatGPT only for language editing and translation assistance. After using this tool, the authors reviewed and edited the content, and the final manuscript was also reviewed by a native English‐speaking language editor. The authors take full responsibility for the content of the publication.

## Supporting information

Supporting File.

## Data Availability

The data that support the findings of this study are available from the corresponding author upon reasonable request.
