# Supplementary material for: Design, Implementation, and Evaluation of an ADDIE Model‐Based Structured Educational Intervention to Improve Anesthesia Students' Patient Handover Skills: A Sequential Explanatory Mixed‐Methods Study
Source: Health Sci Rep. 2026 May 13;9(5):e72528. doi: 10.1002/hsr2.72528 (PMC13172261; doi:10.1002/hsr2.72528)
Supplement: Supplementary file 1 — Supporting File. [file HSR2-9-e72528-s001.docx]

| Domain | Items | Performed | Not Performed |
| --- | --- | --- | --- |
| Patient Demographic Information | Patient demographics (full name, medical record number) |  |  |
| Surgical and Anesthesia Details | Type of surgery and name of surgeon |  |  |
|  | Type of anesthesia and name of anesthesiologist |  |  |
|  | Surgical and anesthesia complications |  |  |
| (Clinical Status and Patient Monitoring) | Patient's underlying diseases and limitations |  |  |
|  | Allergies |  |  |
|  | Vital signs before/after surgery and upon arrival to recovery |  |  |
|  | Patient monitoring in recovery |  |  |
|  | Lines/catheters/foley/drain/IV |  |  |
|  | Administered fluids, IV solutions, and blood products |  |  |
|  | Urine output |  |  |
| (Medication Management and Postoperative Care) | Pain management plan as ordered by surgeon or anesthesiologist |  |  |
|  | Important medications administered in the operating room (ondansetron, dexamethasone, etc.) |  |  |
